# Physiotherapy students’ DiSC behaviour styles can be used to predict the likelihood of success in clinical placements

**DOI:** 10.1186/s12909-019-1825-2

**Published:** 2019-10-17

**Authors:** Nikki Milne, Chanelle Louwen, Dianne Reidlinger, Jo Bishop, Megan Dalton, Linda Crane

**Affiliations:** 1 Faculty of Health Sciences and Medicine, Bond University, Gold Coast, Robina, QLD 4226 Australia; 2 Physiotherapy Department, Logan Hospital, Metro South Health Hospital and Health Service, Meadowbrook, Australia; 3 School of Allied Health, Australian Catholic University, North Sydney, Australia

**Keywords:** Physiotherapy, Clinical education, Behaviour, Assessment, DiSC styles

## Abstract

**Background:**

Behaviour can be defined as the internally coordinated responses (actions or inactions) of whole living organisms (individuals or groups) to internal and/or external stimuli, excluding responses more easily understood as developmental changes. Unlike personality traits, that are thought to be biologically consistent, behaviour, through the application of cognition and reasoning is open to change across time and circumstance, although most humans will display preferred ways of behaving. The objective of this study was to: i) identify the behaviour styles of physiotherapy students and investigate if there is a relationship (predictive or otherwise) between students’ unique behaviour patterns and their clinical placement grades and; ii) examine if this relationship differs when student’s in a Master’s level program as well as student’s in a Bachelor’s level program are explored separately.

**Methods:**

This cross-sectional study with 132 (F = 78, M = 54) physiotherapy students was conducted across two Australian university settings. Measures included Everything DiSC Workplace profile, Assessment of Physiotherapy Practice (APP).

**Results:**

Physiotherapy students (*n* = 133) profiled the following ways: Dominance (D) style *n* = 20 (15%), Influence (i) style *n* = 33 (25%), Steadiness (S) style *n* = 36 (27%) and Conscientiousness (C) *n* = 44 (33%). Students with the individual DiSC styles of i and Conscientiousness / Steadiness (CS) were in the lowest APP quartile for clinical grades and the D style was in the highest quartile. Binary logistic regressions revealed students with an i DiSC style had 3.96 times higher odds, and students with a CS DiSC style had 4.34 times higher odds, of failing a clinical placement. When explored independently, the same trend remained for Master’s level students. Bachelor’s level students with DiSC styles of S and C had failed placements, however these styles were not significantly associated with failure (DiSC S Style: Exp(B) 1.667, *p* = 0.713 (CI: 0.109 to 25.433), DiSC C Style: Exp(B) 11.00, *p* = 0.097 (CI: 0.646 to 187.166)).

**Conclusion:**

Physiotherapy students with DiSC styles i and CS appear to be more likely to fail physiotherapy clinical placements. Further research with larger undergraduate samples is required to establish if relations differ for undergraduate versus postgraduate students.

## Background

Physiotherapy students must demonstrate competence across several clinical areas to successfully complete their program and become registered physiotherapists, and quality learning in clinical settings is critical to their success. In Australia, assessments of clinical competence for physiotherapy students in clinical environments are typically undertaken by health professionals independent of the academic staff of a university, using standardised assessment tools (e.g. Assessment of Physiotherapy Practice (APP)) [1, 2]. Whilst these tools are used to assess competence in areas such as ‘patient assessment’, ‘analysis and planning’, ‘intervention’, ‘evidence-based practice’ and ‘safety’, they also specifically assess items such as ‘professional behaviour’ and ‘communication’ [2].

Anecdotally, clinical educators in the physiotherapy profession have consistently reported that their assessment of a student’s professional behaviour and communication influences their beliefs regarding a student’s clinical competence across other areas / skills. Considering the need for health professionals to demonstrate competence in professional behaviour and communication, a percentage of coursework time is expected to be allocated to development in these areas in health professional programs [3–5]. However outside of mentoring appropriate behaviours, little time is devoted to developing these areas in clinical placement and rather there is a strong expectation from clinical educators that students have basic competence in these areas from the outset of the placement.

The ‘Everything DiSC’ is a standardised and validated tool [6] and was developed to help individuals to better understand their own, and others’, preferred human behaviours and motivators for success across a variety of life settings. The authors of the Everything DiSC [6] propose that by completing the Everything DiSC workplace profile and reflecting on the identified personal drivers for human behaviours, it may be possible for individuals to enhance their communication, rapport and relationships, by modifying their behaviours during short-term interactions with others (e.g. team members, patients, managers) when needed or desired. Communication, rapport building and relationships are critical elements for positive engagement in the workforce [7], learning in a work integrated environment and developing competence in health professionals. For example, if student physiotherapists were aware that they commonly demonstrated behaviour styles consistent with being strongly extroverted and fast-paced and that these behaviour styles unsettled educators in critical care environments, then it may be possible to modify these behaviours through reflection and behaviour state training. Specifically, students may be able to modify their natural tendency to demonstrate these behaviours in that particular setting, despite the displayed behaviour being different to the students’ preferred behaviour style.

Research exploring the relationship between behaviour styles and achievement in clinical placements for health professional students is scarce and is focused on medical students only [8, 9]. Limited empirical literature exists on this topic is despite the allied health professions having strong expectations as documented in their practice thresholds / registration standards [3–5], regarding professional behaviour and communication, of registered health practitioners and student practitioners. It appears that most research in this area has instead focused on personality comparisons between different, and related, professional groups [10–13] rather than behaviour styles in student populations.

There are many different definitions of personality based on proposed underlying theories, however one commonly accepted model of personality which has undergone much research to support its generalisability across various cultures is the Five-factor model (FFM) of personality [14]. The FFM is composed of the following personality trait dimensions: Neuroticism, extraversion, openness to experience, agreeableness and conscientiousness. These personality traits are thought to remain relatively stable over adult life [15, 16] and are believed to have a genetic basis [17], in fact the Five-factor model asserts that personality traits are endogenous biological dispositions, influenced in no way by the environment [14]. Personality in health profession contexts has been examined from perspectives of orientation to person or technique across allied health professions [12]; between occupational therapy and physiotherapy professions [13] and dietitians relative to practice area [10]. Additionally, research has been undertaken to investigate if the personality of students entering a speech-language pathology (SLP) program has changed over time (i.e. changes in personality between cohorts) [11].. Whilst research into personality traits of health professionals is emerging, there is scarce literature referencing behaviour styles of health professional students.

Environmental influences play crucial roles in the functioning of the personality system in several different respects; they shape a vast array of skills, values, attitudes, and identities; which impact the way in which personality traits are expressed and impact the way individuals prefer to behave over time [18]. Whilst behaviour is understood to be influenced by personality and necessarily relies upon internal information processing by an individual (e.g. cognition and endocrine signaling), it can be defined as: the internally coordinated responses (actions or inactions) of whole living organisms (individuals or groups) to internal and/or external stimuli, and excludes responses more easily understood as developmental changes [19]. Behaviour may be represented as a behaviour style [6] or a short term behaviour state [19].

Unlike personality traits, that are thought to be biologically consistent [18], an individual’s behaviour state can, through the application of cognition and reasoning, change across time and circumstance. However, most humans will display consistently preferred ways of behaving (i.e. behaviour styles) [6] unless they cognitively reason the need to change their behaviour state in a given circumstance. To date there has been no study that has investigated physiotherapy students’ preferred behaviour styles, and no research across any of the allied health professions has made comparisons between behaviour styles and clinical placement outcomes.

The physiotherapy education pathway in Australia consists of university programs that range from 2 to 4 years and include both undergraduate (i.e. Bachelor’s) and postgraduate (i.e. Master’s and Extended Master’s) programs. Clinical placements are a key component of all programs and are designed to equip physiotherapy students with the skills and knowledge essential for developing professional competence [20]. Physiotherapy students participate in diverse clinical placements (e.g. cardiorespiratory, neurorehabilitation, musculoskeletal) under supervision of clinical educators across a variety of healthcare settings, providing them with the opportunity to integrate theoretical knowledge into practical skills [21]. The average number of clinical hours for training in Australian physiotherapy education programs is 1000 h [20], with physiotherapy students completing anywhere from four to six, five-week, clinical placements across the course of their program [22]. It is plausible that students completing a Master’s level program may have different preferred behaviour styles than students in a Bachelor’s level program. The additional life experiences of students in Master’s level programs may influence the way student’s behave in clinical practice, potentially impacting their clinical placement outcomes either negatively or positively. Information regarding physiotherapy students’ preferred behaviour styles and how these may impact clinical placement outcomes, may be useful for physiotherapy teaching staff and clinical educators to help inform decisions regarding targeted support strategies to best facilitate desired learning outcomes for students.

Therefore, the aims of this study are to: i) identify the behaviour styles of physiotherapy students and investigate if there is a relationship (predictive or otherwise) between students’ preferred behaviour styles (determined by the Everything DiSC Workplace profile) and their grades in clinical placement experiences (using the APP) and; ii) examine if this relationship differs when students in a Master’s level program as well as students in a Bachelor’s level program are explored separately.

## Methods

### Setting and study design

This cross-sectional study was conducted across two Australian university settings. Ethical approval was obtained by the Bond University Human Research Ethics Committee (Protocol Numbers 16127 and NM03225).

### Recruitment and study participants

**Fig. 1:**
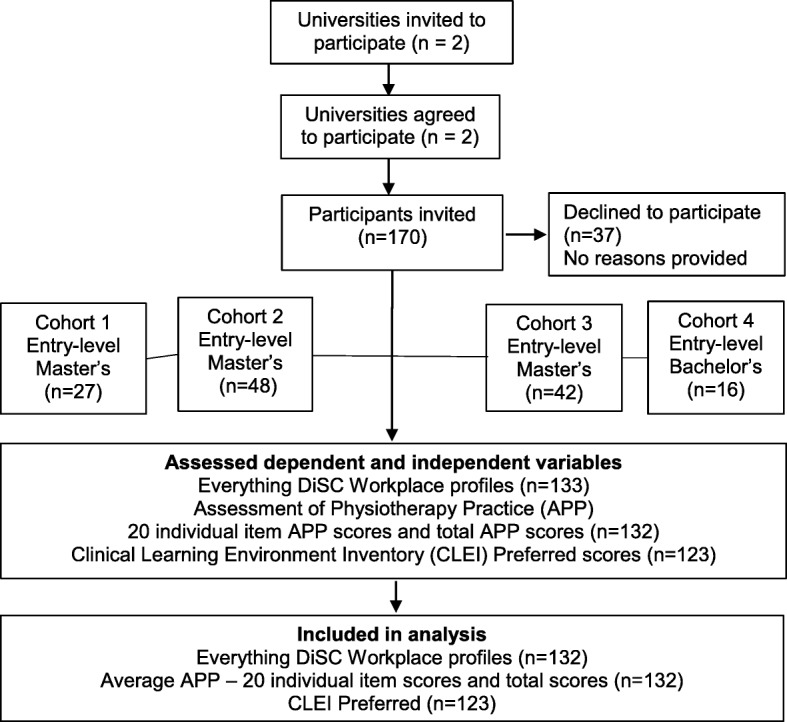
Flow of participants through the study

Australian physiotherapy students based in south-east Queensland were recruited across 2017 (University 1) and 2018 (University 1 and 2). Information sheets and consent forms were sent to all eligible students who were due to undertake clinical placements during the study period. The number of universities included in the study was limited based on funding available to undertake DiSC profiles. All students who were enrolled in the two invited physiotherapy programs and were planning to undertake entry-level physiotherapy placements were eligible to participate. Entry-level placements included any placement across the year, where students who were not yet qualified physiotherapists were for their first time, attempting to reach the minimum entry-level standards to the profession (or above) for a given clinical area (e.g. cardiorespiratory, neurorehabilitation or musculoskeletal). See Fig. 1 for details of the flow of participants through the study. Students in cohorts 1, 2 and 3 were all enrolled in the same Master’s level program in consecutive years of enrolment. Students in cohort 1 had completed their core clinical placements prior to consenting to participate in the study and therefore prior to completing their DiSC profiles. Students in cohort 4 were invited to participate based on the likelihood that they would in the future be allocated to placements at two south-east Queensland hospitals implementing an associated intervention study. All students in cohort 4 were from a Baccalaureate level program in the same geographical region as the first three cohorts.

### Predictors, outcome measures and covariates

#### Everything DiSC - workplace (predictor)

**Fig. 2:**
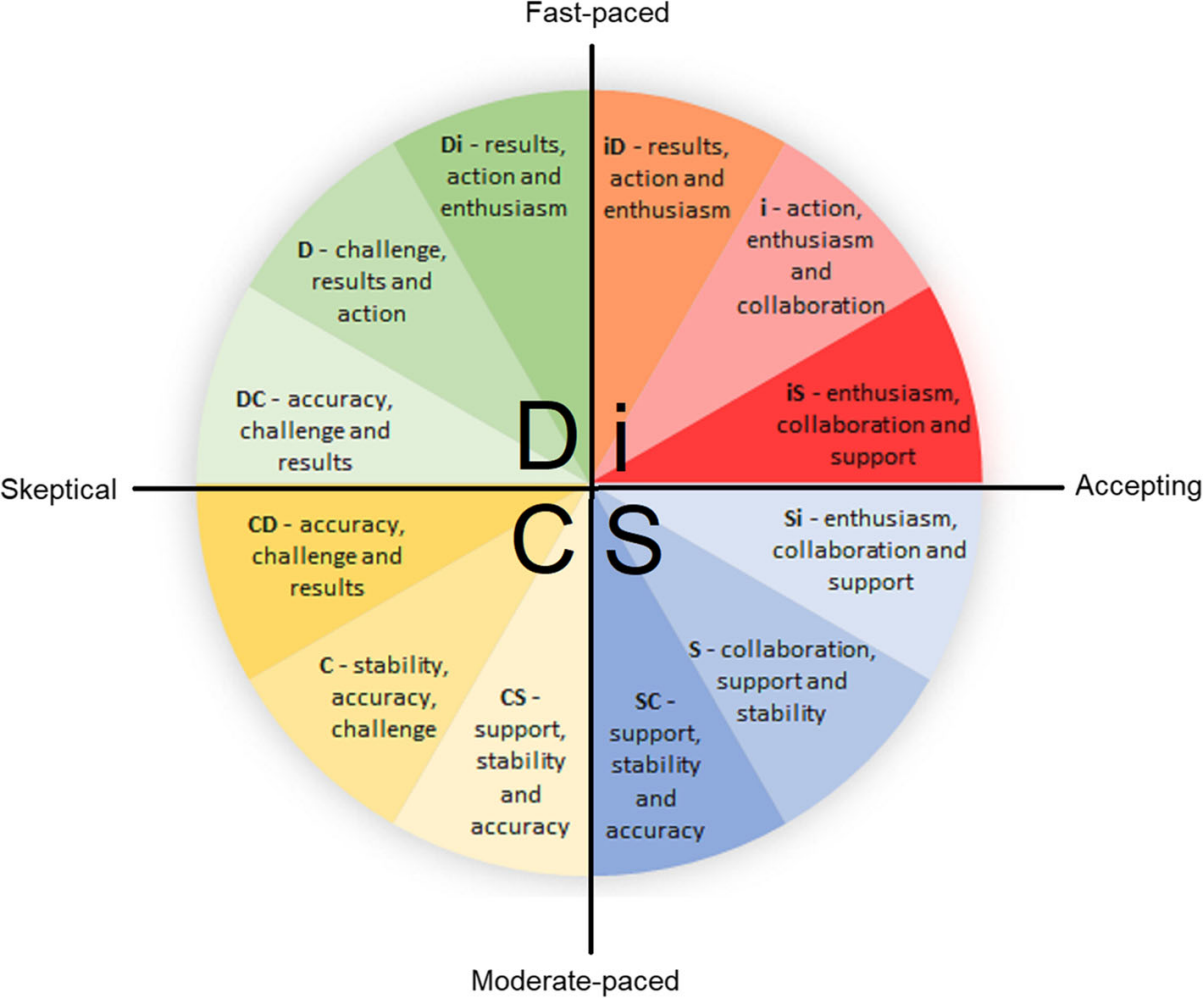
DiSC styles and priorities driving behaviour with 2-dimensional scales

The Everything DiSC - Workplace is a valid and reliable personal development assessment that takes approximately 15–20 min to complete using an online platform and measures an individual’s behaviour styles, tendencies and priorities across two main dimensions; i) Fast-paced versus moderate-paced and ii) Skeptical versus accepting [6] (see Fig. 2). In addition to the above-mentioned dimensions, eight main DiSC scales (i.e. angle points within a circumplex) were initially identified in the development of the Everything DiSC; Di/iD, i, iS/Si, S, SC/CS, C, CD/DC, D. The clinometric value of the Everything DiSC was evaluated during its development using these scales (angle points) as measures of various behaviour tendencies. The scales on the Everything DiSC instrument demonstrate good-to-excellent internal consistency with each individual scale measuring above 0.83 using Cronbach’s Alpha. The test-retest reliability of the Everything DiSC scales within a two-week period is reported to be above 0.85 [6]. After assessing the clinometric value of each angle point within the 360 degree circumplex, four main styles (Dominance (D), Influence (i), Steadiness (S), Conscientiousness (C)) were identified across four quarters with twelve individual sub-styles each occupying 30 degrees of the circumplex to represent different behaviour styles. The four main styles (D, i, S and C) and the twelve individual sub-styles and their corresponding priorities driving behaviour are displayed graphically in Fig. 2.

The median angle change for repeated measures of the Everything DiSC with the same participant is approximately 12 degrees and those with a stronger inclination towards a given DiSC style are likely to show greater consistency over repeated measures [6].

On the 2-dimensional scales of the DiSC, the Dominance / Influence styles (Di or iD) are reported to be fast-paced and most commonly reported as ‘dynamic’. The Influence style (i) is fast-paced and accepting, most commonly reported as ‘lively and outgoing’. The Influence / Steadiness (iS or Si) styles are accepting and most commonly reported as ‘cheerful’. The Steadiness (S) style is reported to be moderate-paced and accepting and most commonly referred to as ‘gentle’. The Steadiness / Conscientiousness (SC or CS) styles are moderate paced and ‘softly spoken’. The Conscientiousness (C) style is reported to be moderate-paced and skeptical and most commonly referred to as ‘analytical’. The Conscientiousness / Dominance (CD or DC) style is skeptical and commonly referred to as ‘challenging’. The Dominance (D) style is fast-paced and skeptical and most commonly referred to as ‘strong-willed’.

The DiSC has a connection to other models of psychometric theory, in particular the introversion / extraversion construct of the five-factor model (FFM) of personality [14], where the extraversion factor (a personality trait) of the FFM runs diagonally through the DiSC circumplex, at approximately 45 degrees to the right from the vertical line intersecting the Influence (i) quadrant, with the introversion factor (the opposite personality trait) intersecting the Conscientiousness (C) quadrant of the DiSC circumplex. In terms of validity, the DiSC model proposes that adjacent DiSC styles (e.g. Di and i) will have moderate positive correlations and that styles that are theoretically opposite (e.g. i and C) should have strong negative correlations and evidence of such correlations for construct and criterion validity to support this proposition are provided in the Everything DiSC manual [6]. The alignment of the introversion / extraversion construct suggests that the i and C styles and their adjacent sub-styles may be more strongly influenced by these personality traits than the D and S styles.

### Assessment of physiotherapy practice (APP) (outcome)

Clinical performance during all clinical placements was measured using the APP [1, 2], which is a valid [23] and reliable [24] instrument for quantifying clinical performance during physiotherapy placements occurring over 5 weeks or more. The APP is used to assess the observable behaviour attributes displayed by the student across 20 items covering seven domains of clinical performance: 1. Professional behaviour; 2. Communication; 3. Assessment; 4. Analysis and Planning; 5. Intervention; 6. Evidence-based Practice and; 7. Risk Management. Each individual item is scored 0–4, with 0 = infrequently / rarely demonstrating performance indicators, through to 4 = demonstrating most performance indicators to an excellent standard. The maximum total score for the APP is 80 and each student’s scores were converted to a percentage score for analysis in this study. Additionally, the APP has a final global rating scale of ‘Not Adequate’ = 1, ‘Adequate’ = 2, ‘Good’ = 3 and ‘Excellent’ = 4. Students commenced their clinical placements after completing the relevant pre-clinical coursework for the placements and the clinical educators scoring the APP were registered physiotherapists in Australia who had completed training on the standardised use of the APP or were supervised by a senior clinical educator who had undertaken the standardised training. End of unit APP scores were collected and analysed for this study. The APP scores analysed in this study were from students undertaking clinical placements in the following areas: cardiorespiratory, orthopaedics, musculoskeletal outpatients, neurological rehabilitation, paediatrics, women’s health and mixed placements. Depending on the time of year that students were recruited to the study, between two and six placements were completed by each student. Students in the Master’s level program participated in placements ranging from their first to their sixth clinical placement, whilst students in the Bachelor’s level program participated in placements ranging from their second to their sixth clinical placement. With no published reference data available to investigate change in APP scores over consecutive placements, the global rating scale scores from three consecutive cohorts from the participating Master’s level program were inspected prior to analysing the data for this study. This inspection of data showed that the cohort who had only completed two clinical placements had very similar APP global rating scores (Average: 2.35 / 4) to the cohorts who had completed five or six clinical placements (Average: 2.34 / 4), suggesting that students did not have comparatively higher grades on their later completed placements. This consistency in average scores (despite the number of placements completed) is likely attributable to each individual clinical area (e.g. cardiorespiratory, neurorehabilitation etc.) having unique expectations for achieving clinical competence. Subsequent to this earlier inspection of data, students’ APP scores were averaged for individual items and total scores for analysis in this study.

### Clinical learning environment inventory (CLEI) – preferred form (potential confounder)

Physiotherapy clinical education takes place in environments known to be complex social atmospheres where the educator is required to monitor clients’, students’, and clinicians’ needs. Students may have different expectations regarding the clinical learning environment, and this may impact their interactions with the clinical educator, patients / clients and peers during the placement. Consequently, this may impact their standard of performance during the placement and subsequent assessment on the APP. For this reason the expectations regarding the clinical learning environment were recorded using the CLEI preferred form; a valid and reliable assessment of students perceived preferred learning environment in clinical education contexts [25]. Whilst the CLEI includes components representative of individualisation, innovation, involvement, personalisation and task orientation, only the total combined score is explored in this study as a possible confounder on the relationship between the DiSC and APP. A total CLEI score was derived by summing all CLEI positive items and summing all CLEI negative items before deducting the total negative item score from the positive item score. The CLEI was used to determine the expectations of the student regarding their clinical education experiences prior to placement as this was considered a possible confounder on the relationship between the individual DiSC styles and APP scores.

### Covariates

Gender, student cohort (see Fig. 1) and university degree level (i.e. entry-level Master’s versus entry-level Bachelor’s level programs) were identified and recorded as possible covariates. CLEI preferred responses (total scores) were considered a possible confounder in the relationships between the DiSC styles and APP total scores.

#### Procedure

Everything DiSC Workplace assessments were undertaken by a member of the research team who was licensed to administer the Everything DiSC Workplace profiles. All students were provided with a detailed report regarding their Everything DiSC style and preferred behaviours and were given a 1-h debriefing either in a group setting or individually (depending on preference) about their individual DiSC style and other DiSC styles. Cohort 1 completed six clinical placements and did not receive their profile until after all analysed placements were complete. Cohort 2 completed six clinical placements and received their debriefing after their fourth clinical placement. Cohort 3 completed two clinical placements during the study period and were provided their individual DiSC profiles prior to starting their first placement. Cohort 4 completed six clinical placements during the study period but were only offered their DiSC profiles as they entered an intervention arm of a related study between their 2nd and 6th placements. All students were asked to complete the CLEI Preferred form [25] at the time of recruitment. Participants undertook two-to-six, five-week placements across the study period depending on the timing of recruitment. APP results were provided to each participant by their clinical educator before they were provided to the university. At the completion of the study period the APP results for each participant were recorded in SPSS (Version 24) [26] and averaged for individual item scores and total end of unit scores prior to analysis.

#### Analysis of data

All statistical analyses were conducted using the Statistical Package for the Social Sciences (Version 24) [26]. Descriptive statistics (means, standard deviations (SD), medians, range, percentages and frequencies) were calculated to characterise the participants in the study. Additionally, APP Total Score quartile ranges were calculated, as were the APP scores for students in each DiSC style group and these were attributed to the relevant APP quartile. Assumptions for parametric statistics were explored using normality of data measures including frequency distributions and equality of variances. To explore possible confounders to the primary analyses, Independent samples t-tests (using Levene’s Test for Equality of Variances) were used to determine if differences existed in APP total scores between males and females. Additionally, one-way ANOVA was used to determine if differences in APP average scores existed between the cohorts of students recruited to the study. The relationship between CLEI Preferred total scores and APP total scores was investigated using Pearson’s Product-Moment Correlations to determine if the CLEI could be a possible confounder in the relationship between DiSC styles and APP scores. Differences in APP total scores for students in individual DiSC style groups were analysed using Kruskal Wallis tests (with Mann Whitney U post hoc analyses) after tests for assumptions of normality and equal distribution of APP data were not met between DiSC style groups. Prior to exploring the likelihood of students with a particular DiSC style failing a placement, students were dichotomized into the following groups; “no fails” and “one or more fail”. Binary logistic regressions were used to explore how likely students with a particular DiSC profile were to fail a placement compared to students in the total combined population within the study. Additionally, Binary logistic regressions exploring the same relationships were undertaken separately for students in the entry-level Master’s level program compared to students in the entry-level Bachelor’s level program. Significance level was set at *p* = .05 unless otherwise stated.

## Results

### Participants

A total of 133 physiotherapy students across 4 cohorts from two entry-level physiotherapy (1 Masters level, 1 Bachelor) programs in Australia consented to participate in the study. Data were collected from all 133 students, however one student withdrew from their physiotherapy program of study prior to undertaking clinical placements and consequently data from this student was not included in analysis leaving 132 (Female = 78, Male = 54) remaining in the study. Most (*n* = 117, 88.63%) of the students participating in this study were enrolled in an entry-level Master’s physiotherapy program, whilst the others (12.12% (*n* = 16) were enrolled in an entry-level Bachelor’s physiotherapy program. Figure 1 provides a summary of the flow of participants through the study. Students were represented across each of the global DiSC styles in the following distributions: Dominance (D) = 20 (15%), Influence (i) = 33 (25%), Steadiness (S) = 36 (27%) and Conscientiousness (C) = 44 (33%). The distribution of student’s global DiSC styles across the study demonstrates that over half of students (60%) were at the moderate-paced end of the vertical (fast-paced versus moderate-paced) continuum and there was an almost equal distribution of students at each end of the horizontal (skeptical versus accepting) continuum. To characterise the study participants the mean (+ − SD) total APP scores for the total group, and by gender and program level, are provided in Table 1. Twelve percent of students (*n* = 16) across the combined cohorts failed at least one clinical placement.

**Table 1:** Mean scores and standard deviations for APP results for total group, and gender and program level

APP Scores (Averaged across all clinical placements)	Combined (*n* = 132) Mean (SD)	Males (*n* = 55) Mean (SD)	Females (*n* = 78) Mean (SD)	Master’s level (*n* = 117) Mean (SD)	Bachelor’s level (*n* = 16) Mean (SD)
Professional Behaviour					
1.Demonstrates an understanding of client rights and consent	3.68 (0.39)	3.65 (0.48)	3.71 (0.31)	3.67 (0.40)	3.77 (0.28)
2.Demonstrates commitment to learning	3.57 (0.49)	3.45 (0.60)	3.66 (0.39)	3.55 (0.51)	3.71 (0.30)
3.Demonstrates ethical, legal & culturally responsive practice	3.64 (0.45)	3.56 (0.55)	3.70 (0.36)	3.63 (0.47)	3.71 (0.29)
4.Demonstrates collaborative practice	3.33 (0.55)	3.29 (0.58)	3.35 (0.53)	3.33 (0.57)	3.33 (0.35)
Communication					
5.Communicates effectively and appropriately - Verbal/Non-verbal	3.29 (0.62)	3.23 (0.65)	3.33 (0.60)	3.28 (0.64)	3.37 (0.48)
6.Demonstrates clear and accurate documentation	3.31 (0.58)	3.14 (0.65)	3.42 (0.50)	3.29 (0.61)	3.45 (0.35)
Assessment					
7.Conducts an appropriate client-centred interview	3.31 (0.53)	3.22 (0.58)	3.37 (0.48)	3.31 (0.54)	3.32 (0.44)
8.Selects and measures relevant health indicators and outcomes	3.04 (0.55)	2.98 (0.64)	3.08 (0.47)	3.02 (0.57)	3.12 (0.33)
9.Performs appropriate physical assessment procedures	3.17 (0.54)	3.14 (0.56)	3.20 (0.53)	3.17 (0.55)	3.17 (0.52)
Analysis & Planning					
10.Appropriately interprets assessment findings	3.05 (0.61)	2.99 (0.67)	3.09 (0.56)	3.04 (0.62)	3.10 (0.47)
11.Identifies and prioritises client’s problems	3.10 (0.60)	3.07 (0.65)	3.13 (0.58)	3.09 (0.63)	3.22 (0.40)
12.Sets realistic short- and long-term client-centred goals	2.98 (0.55)	2.91 (0.62)	3.04 (0.49)	2.97 (0.56)	3.12 (0.39)
13.Selects appropriate intervention in collaboration with the client	3.10 (0.58)	3.02 (0.63)	3.16 (0.54)	3.09 (0.61)	3.19 (0.34)
Intervention					
14.Performs interventions appropriately	3.25 (0.60)	3.20 (0.64)	3.28 (0.56)	3.24 (0.62)	3.31 (0.44)
15.Is an effective educator	3.20 (0.63)	3.17 (0.71)	3.22 (0.58)	3.19 (0.65)	3.26 (0.50)
16.Monitors the effect of intervention	3.15 (0.58)	3.10 (0.62)	3.18 (0.55)	3.13 (0.60)	3.25 (0.37)
17.Progresses intervention appropriately	3.11 (0.57)	3.08 (0.61)	3.13 (0.55)	3.11 (0.60)	3.07 (0.36)
18.Undertakes discharge planning	2.98 (0.52)	2.92 (0.60)	3.03 (0.45)	2.97 (0.53)	3.10 (0.39)
Evidence-based Practice					
19.Applies evidence-based practice in client-centred care	3.20 (0.54)	3.09 (0.61)	3.27 (0.46)	3.19 (0.55)	3.22 (0.44)
Risk Management					
20.Identifies adverse events/near misses and minimises risk associated with assessment and interventions	3.32 (0.61)	3.24 (0.55)	3.37 (0.55)	3.31 (0.63)	3.34 (0.45)
Total APP out of 80	64.78 (9.99)	63.41 (11.45)	65.73 (8.79)	64.59 (10.35)	66.14 (6.97)
Total APP Percentage (%)	80.95 (12.48)	79.21 (14.30)	82.16 (10.99)	80.71 (12.93)	82.68 (8.70)

*SD* Standard Deviation, *APP* Assessment of Physiotherapy Practice. Each individual APP item is scored 0–4, with 0 = infrequently / rarely demonstrates performance indicators, through to 4 = demonstrates most performance indicators to an excellent standard

Each participants’ APP Total score was averaged across their placements and this information was used to create APP Total score quartile ranges. After the quartile ranges were created, each of the DiSC styles were allocated into APP quartiles according to the average APP total score calculated for students within each DiSC style (see Table 2).

**Table 2:** APP Total Score Quartile Ranges and Corresponding DiSC Styles

APP Total Score Quartile ranges	Corresponding average APP Total Score range	DiSC styles that fell within the identified APP Quartile range. (n, %)
1st Quartile = < 24.9%ile	< 77.65%	i (13, 9.85%) CS (16, 12.12%)
2nd Quartile = 25–49.9%ile	77.66–83.36%	DC (6, 4.55%) Di (7, 5.30%) iD (5, 3.79%) S (20, 15.15%) SC (10, 7.58%) C (22, 16.67%)
3rd Quartile = 50–74.9%ile	83.37–88.95%	iS (15, 11.36%) CD (5, 3.79%) Si (6, 4.55%)
4th Quartile = > 75%ile	> 88.95%	D (7, 5.30%)

**Fig. 3:**
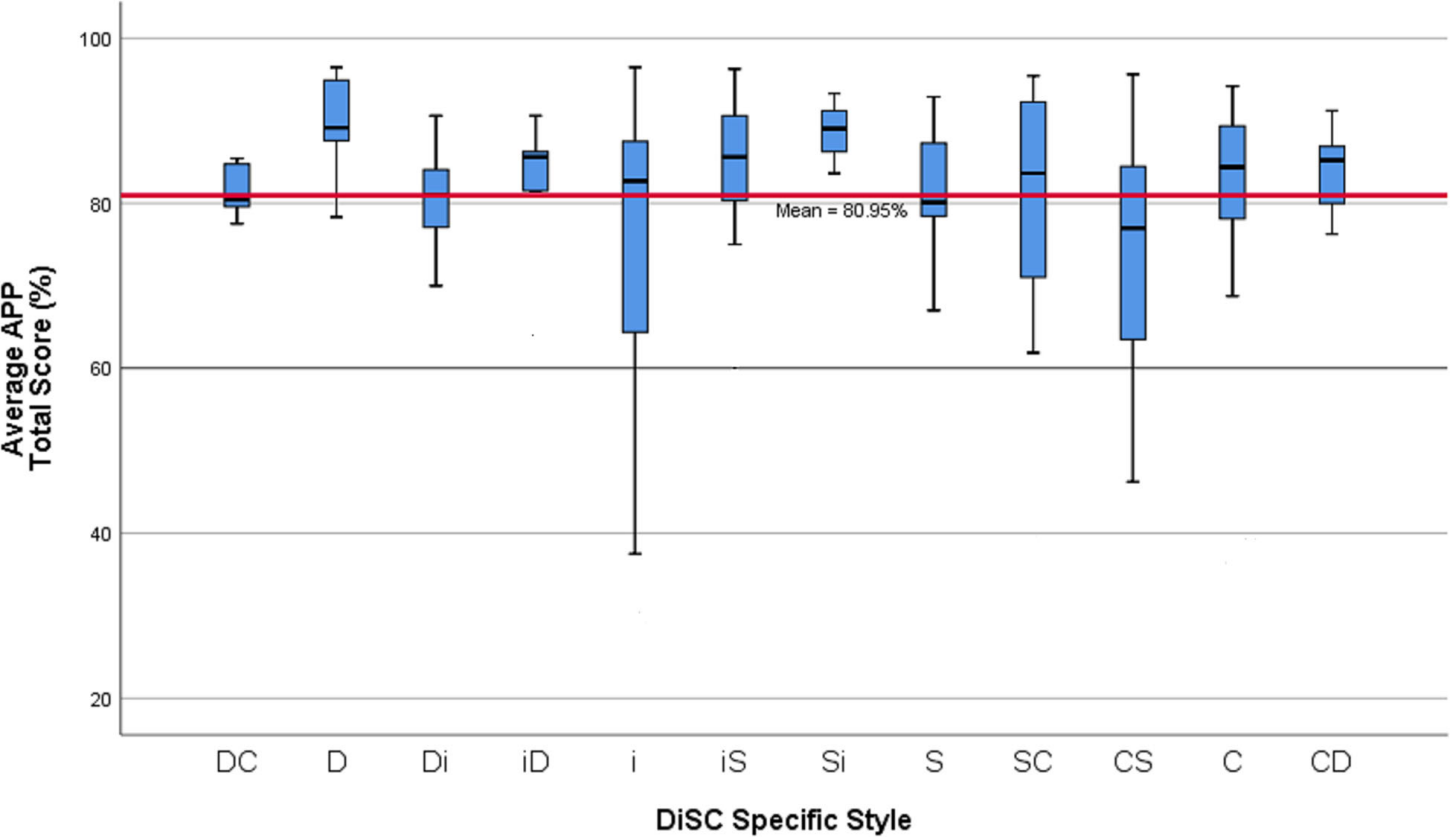
Average APP Total Scores according to individual DiSC Styles for the Total Group

One-way ANOVA revealed no significant differences in average APP total scores between different cohorts of students (F (3,131) = 1.878, *p* = 0.137) including those from the entry-level Master’s program compared to participants in the entry-level Bachelor’s program. Additionally, Independent samples t-tests revealed no significant differences in APP total scores between males and females (F = 1.199, t = 1.34, DF = 130, *p* = 0.183) and consequently the APP data was aggregated for subsequent analyses. Pearson’s correlations revealed no significant relationship between the CLEI-Preferred total score and the APP Total score (r = 0.039, *p* = 0.668) and the CLEI was therefore not included as a contributing confounder in further analyses exploring relationships between the APP scores and DiSC styles. Figure 3 provides a graphical representation of the various distributions of average APP Total scores grouped by individual DiSC styles.

As APP total score distributions were not considered to meet the assumptions for normality and equal distribution, differences in students’ mean APP scores (mean ranks) between individual DiSC style groups were analysed using Kruskal Wallis H tests and Mann Whitney U post hoc analyses. Several differences were identified, and these results are provided in Table 3. Post hoc analyses revealed statistically significant differences between students with the D and CS DiSC styles in the APP item – *Demonstrates ethical, legal and culturally responsive practice*. Statistically significant differences were also noted between students with the D and CS DiSC styles and students with the Si and CS styles for the APP item – *Progresses intervention appropriately*. Whilst many other differences were identified across APP item scores, after accounting for the number of groups being analysed to modify the significance value to *p* = 0.004, no further items were identified as having significant differences (see Table 3).

**Table 3:** Mean APP scores for each individual DiSC style and differences between DiSC styles

	APP Mean Scores (averaged across all clinical placements) for Individual Everything DiSC Style Groups *n* = 132 students Mean (SD)													
APP Items	1. DC *n* = 6	2. D *n* = 7	3. Di n = 7	4. iD n = 5	5. i *n* = 13	6. iS *n* = 15	7. Si *n* = 6	8. S n = 20	9. SC *n* = 10	10. CS n = 16	11. C *n* = 22	12. CD *n* = 5	Kruskal Wallis Difference between DiSC Styles (*p*-value)	Mann Whitney U Post Hoc Differences between DiSC Styles (*p*-value)
Professional Behaviour														
1.Demonstrates an understanding of client rights and consent	3.73 (0.15)	3.95 (0.08)	3.83 (0.19)	3.86 (0.22)	3.45 (0.69)	3.78 (0.19)	3.81 (0.25)	3.71 (0.28)	3.60 (0.62)	3.50 (0.36)	3.65 (0.39)	3.87 (0.22)	0.075	
2.Demonstrates commitment to learning	3.50 (0.19)	3.78 (0.21)	3.50 (0.30)	3.62 (0.41)	3.19 (0.86)	3.74 (0.33)	3.81 (0.19)	3.58 (0.32)	3.45 (0.82)	3.50 (0.49)	3.68 (0.40)	3.50 (0.47)	0.228	
3.Demonstrates ethical, legal & culturally responsive practice	3.58 (0.15)	3.93 (0.13)	3.76 (0.25)	3.81 (0.21)	3.28 (0.88)	3.82 (0.23)	3.81 (0.27)	3.71 (0.22)	3.55 (0.65)	3.45 (0.38)	3.66 (0.42)	3.70 (0.45)	0.040*	D – CS: *p* = 0.001* D – i: *p* = 0.019 Si – CS = 0.040
4.Demonstrates collaborative practice	3.36 (0.25)	3.67 (0.30)	3.17 (0.37)	3.39 (0.38)	3.17 (0.84)	3.57 (0.43)	3.58 (0.09)	3.34 (0.41)	3.25 (0.75)	3.00 (0.67)	3.34 (0.56)	3.43 (0.25)	0.108	
Communication														
5.Communicates effectively and appropriately - Verbal/Non-verbal	3.29 (0.27)	3.78 (0.31)	3.14 (0.66)	3.34 (0.23)	3.05 (0.87)	3.63 (0.31)	3.64 (0.31)	3.31 (0.40)	3.03 (0.92)	2.96 (0.77)	3.28 (0.59)	3.53 (0.40)	0.030*	D – CS: p = 0.01 D – i: p = 0.019 Si – CS: *p* = 0.059 Si – i: *p* = 0.179
6.Demonstrates clear and accurate documentation	3.43 (0.16)	3.63 (0.29)	3.33 (0.54)	3.08 (0.53)	2.90 (0.89)	3.46 (0.38)	3.72 (0.39)	3.30 (0.35)	3.28 (0.74)	3.16 (0.79)	3.36 (0.54)	3.30 (0.45)	0.269	
Assessment														
7.Conducts an appropriate client-centred interview	3.12 (0.25)	3.72 (0.30)	3.45 (0.45)	3.49 (0.40)	3.00 (0.89)	3.47 (0.31)	3.72 (0.20)	3.29 (0.37)	3.27 (0.67)	3.06 (0.55)	3.30 (0.52)	3.40 (0.42)	0.050	
8.Selects and measures relevant health indicators and outcomes	3.11 (0.29)	3.29 (0.23)	3.07 (0.35)	2.95 (0.57)	2.64 (0.84)	3.21 (0.49)	3.36 (0.19)	3.11 (0.38)	2.99 (0.63)	2.72 (0.63)	3.12 (0.52)	3.17 (0.41)	0.122	
9.Performs appropriate physical assessment procedures	3.15 (0.28)	3.43 (0.45)	3.07 (0.38)	3.43 (0.57)	2.92 (0.69)	3.35 (0.38)	3.39 (0.29)	3.22 (0.47)	3.05 (0.78)	2.95 (0.58)	3.14 (0.59)	3.47 (0.36)	0.379	
Analysis & Planning														
10.Appropriately interprets assessment findings	3.09 (0.27)	3.39 (0.36)	3.19 (0.48)	3.15 (0.74)	2.62 (0.90)	3.14 (0.45)	3.42 (0.25)	3.10 (0.39)	2.93 (0.69)	2.70 (0.71)	3.15 (0.62)	3.30 (0.45)	0.108	
11.Identifies and prioritises client’s problems	3.24 (0.10)	3.44 (0.39)	3.07 (0.25)	3.15 (0.42)	2.78 (0.93)	3.24 (0.47)	3.33 (0.38)	3.18 (0.30)	3.03 (0.78)	2.72 (0.77)	3.23 (0.66)	3.17 (0.42)	0.346	
12.Sets realistic short- and long-term client-centred goals	3.12 (0.26)	3.39 (0.30)	2.83 (0.45)	2.90 (0.55)	2.73 (0.82)	3.12 (0.43)	3.36 (0.19)	2.99 (0.33)	2.98 (0.67)	2.76 (0.62)	2.97 (0.64)	3.10 (0.25)	0.189	
13.Selects appropriate intervention in collaboration with the client	3.10 (0.20)	3.59 (0.32)	3.02 (0.38)	3.14 (0.42)	2.69 (0.87)	3.37 (0.44)	3.31 (0.29)	3.17 (0.37)	2.98 (0.72)	2.84 (0.73)	3.13 (0.60)	3.27 (0.25)	0.053	
Intervention														
14.Performs interventions appropriately	3.26 (0.18)	3.60 (0.39)	3.10 (0.40)	3.43 (0.57)	2.94 (0.90)	3.40 (0.36)	3.69 (0.27)	3.34 (0.41)	3.08 (0.87)	3.03 (0.68)	3.21 (0.65)	3.43 (0.09)	0.177	
15.Is an effective educator	3.21 (0.23)	3.69 (0.38)	3.19 (0.56)	3.09 (0.37)	2.91 (0.96)	3.42 (0.35)	3.69 (0.27)	3.19 (0.40)	3.08 (0.83)	2.84 (0.84)	3.22 (0.62)	3.57 (0.25)	0.024*	D - CS: *p* = 0.012 D – i: *p* = 0.030 Si – CS: *p* = 0.017 Si – i: *p* = 0.029
16.Monitors the effect of intervention	3.15 (0.37)	3.44 (0.31)	3.26 (0.43)	2.99 (0.58)	2.78 (0.87)	3.36 (0.47)	3.47 (0.27)	3.16 (0.37)	3.18 (0.78)	2.86 (0.77)	3.21 (0.51)	3.17 (0.20)	0.261	
17.Progresses intervention appropriately	3.12 (0.23)	3.58 (0.35)	3.29 (0.47)	3.10 (0.42)	2.76 (0.92)	3.24 (0.39)	3.50 (0.18)	3.19 (0.41)	2.99 (0.66)	2.74 (0.61)	3.14 (0.61)	3.20 (0.22)	0.032*	D - CS: p = 0.001* D – i: *p* = 0.024 Si – CS: *p* = 0.002* Si – i: *p* = 0.036
18.Undertakes discharge planning	3.01 (0.22)	3.38 (0.40)	2.90 (0.32)	3.04 (0.62)	2.73 (0.80)	3.06 (0.34)	3.22 (0.20)	3.03 (0.34)	2.94 (0.62)	2.78 (0.62)	2.99 (0.57)	3.10 (0.42)	0.542	
Evidence-based Practice														
19.Applies evidence-based practice / client-centred care	3.15 (0.17)	3.48 (0.29)	3.10 (0.35)	3.05 (0.67)	2.88 (0.90)	3.45 (0.36)	3.58 (0.25)	3.23 (0.43)	3.21 (0.59)	3.06 (0.53)	3.19 (0.54)	3.07 (0.52)	0.227	
Risk Management														
20.Identifies adverse events/near misses, minimises risk associated with assessment and interventions	3.34 (0.25)	3.73 (0.27)	3.17 (0.35)	3.33 (0.21)	3.04 (0.95)	3.46 (0.43)	3.81 (0.16)	3.44 (0.42)	3.26 (0.87)	2.97 (0.73)	3.29 (0.63)	3.40 (0.43)	0.030*	D - CS: *p* = 0.008 D – i: p = 0.019 S – CS: *p* = 0.021 Si – i: *p* = 0.046
Total APP out of 80	65.08 (2.48)	71.89 (5.09)	64.45 (5.43)	65.34 (8.16)	58.46 (16.67)	68.29 (5.58)	71.22 (2.55)	65.55 (5.76)	63.13 (14.11)	59.60 (12.04)	65.28 (10.11)	67.13 (4.70)	0.090	
Total APP Percentage (%)	81.35 (3.10)	89.86 (6.36)	80.57 (6.78)	81.68 (10.20)	73.08 (20.84)	85.37 (6.97)	88.76 (3.61)	81.94 (7.20)	78.91 (17.64)	74.50 (15.05)	81.53 (12.62)	83.92 (5.88)	0.090	

Kruskal Wallis Significance (*p* = 0.05)

Mann Whitney U Significance value was divided by the number of DiSC Styles (*p* = 0.05 / 12 = 0.0042)

Binary logistic regressions were used to explore how likely students with a particular DiSC style were to fail a placement compared to the total student sample within the study. A statistically significant relationship was found between the DiSC styles of i and CS and the likelihood of receiving a fail grade on the APP. As the 95% CI did not overlap, we can conclude that compared to the total student sample, students with an i DiSC style had 3.96 times higher odds, and students with a CS DiSC style had 4.34 times higher odds, of failing a placement during their clinical placement program (see Table 4).

**Table 4:** Binary Logistic Regressions and odds of a student failing a clinical placement based on their DiSC style

DiSC Style	B	S.E	Wald	df	Sig.	Exp(B)	95% CI for EXP (B)	
							Lower	Upper
DC	−19.275	16,408.711	0.000	1	0.999	0.000	0.000	–
D	−19.284	15,191.515	0.000	1	0.999	0.000	0.000	–
Di	−19.284	15,191.515	0.000	1	0.999	0.000	0.000	–
iD	−19.266	17,974.843	0.000	1	0.999	0.000	0.000	–
i	1.377	0.674	4.178	1	0.041*	3.963	1.058	14.840
iS	−19.360	10,377.780	0.000	1	0.999	0.000	0.000	–
Si	0.392	1.129	0.120	1	0.729	1.480	0.162	13.541
S	−1.013	1.064	0.907	1	0.341	0.363	0.045	2.922
SC	0.657	0.840	0.611	1	0.434	1.929	0.372	10.007
CS	1.468	0.626	5.504	1	0.019*	4.339	1.273	14.786
C	0.228	0.690	0.109	1	0.741	1.256	0.325	4.857
CD	−19.266	17,974.843	0.000	1	0.999	0.000	0.000	–

Significance set at *p* = 0.05; B - Unstandardised regression weight; *S. E* Standard Error, *Wald* statistical test for individual predictor variable, *df* degrees of freedom, *Sig*. Significance level, *Exp(B)* Predicted odds ratio, *CI* Confidence Interval

Binary logistic regression was also used to explore the same relationships with students in the Master’s level program compared to the Bachelor’s level program. When explored separately, students with DiSC styles i (Exp(B) 4.642, *p* = 0.027 (CI: 1.189 to 18.121)) and CS (DiSC CS Style: Exp(B) 7.422, *p* = 0.003 (CI: 1.963 to 28.064)) remained significantly associated with failing a placement for students in the post-graduate program. No further significant relationships were exposed. When undergraduate students were explored independently, only students with the DiSC styles of S and C had failed placements, however these styles were not significantly associated with failing a placement for undergraduate students (DiSC S Style: Exp(B) 1.667, *p* = 0.713 (CI: 0.109 to 25.433), DiSC C Style: Exp(B) 11.00, *p* = 0.097 (CI: 0.646 to 187.166)) nor were they significant predictors for the total student population in this study.

## Discussion

This study aimed to identify the behaviour styles of physiotherapy students and to investigate the relationship between students’ behaviour styles (as measured by the Everything DiSC profiles) and their grades during clinical placement experiences. Additionally, we aimed to examine if this relationship differs when student’s in a Master’s level program as well as student’s in a Bachelor’s level program are explored separately. Physiotherapy students’ behaviour styles and their ability to predict success on placement has not been investigated previously. Prior to this study, it was unclear if there was a relationship between physiotherapy students’ behaviour styles and clinical placement outcomes, irrespective of program level.

### Characterising physiotherapy students behaviour styles using everything DiSC

The present study is the first of its kind to profile physiotherapy students across both undergraduate and postgraduate programs using the ‘Everything DiSC’ workplace instrument. Although this study demonstrated that all DiSC styles are present across the physiotherapy student cohorts, results demonstrated that the Conscientiousness (C) style (33%) was most prevalent. There was a higher percentage (60%) of physiotherapy students taking up the bottom two quarters of the DiSC circumplex (i.e. demonstrating C and S DiSC styles). These findings suggest that a larger proportion (60%) of physiotherapy students in this study demonstrate moderate-paced behaviours, with lower levels of outward energy and typically display more internal reflective behaviour [6].

Whilst there is no comparison literature for physiotherapists, this distribution of DiSC styles differed somewhat to the findings published by Mun and Hwang [27] about nurses whose preferred behaviour styles were shown to be Dominance (11.4%), Influence (42%), Steadiness (29%), and Conscientiousness (17.6%), with the distribution of behaviour styles in the bottom two quarters of the DiSC (S and C) accounting for less than half (46.6%) of nurses and the most prevalent style being Influence (i). These findings suggest that nurses more commonly demonstrate a higher outward energy and tend to me more outspoken, assertive and fast-paced [6]; behaviour styles that were associated with increased likelihood of failure for physiotherapy students in the present study.

Although the results of the present study show the mean total APP scores for all styles met a passing standard, physiotherapy students with the Dominance (D) style achieved the highest APP total percentage scores compared to the Influence (i) and Conscientiousness / Steadiness (CS) styles which achieved the lowest APP total percentage scores (see Table 2). In the opinion of the authors, clinical education can be an emotionally and physically demanding experience for some students and requires considerable persistence and self-directed study to successfully complete clinical placements. A recent study of medical students’ behaviour traits [28] found that students who were characterised as having very high persistence and self-directedness, were more strongly indicative of displaying well-being and resilience. When the DiSC was developed the terms persistence and self-directedness were commonly used to describe persons with a Dominance (D) behaviour style [6] and this may be one of the reasons physiotherapy students with a D style in our study achieved the highest APP scores.

### Relationship between DiSC style and APP results

The findings of the present study suggest that physiotherapy students with Influence (i) and Conscientiousness / Steadiness (CS) styles were associated with significantly greater likelihood of failing a placement during their clinical placement program. Students with an i style tend to talk more than other styles and function more impulsively than the average person, exhibiting priorities of enthusiasm, action and collaboration [6]. Whilst these traits can create very successful outcomes in some circumstances, it is these traits when under pressure that may lead to students with an i style appearing more disorganised, overly expressive and making quick progressions without considering consequences [29]; attributes that clinical educators are likely to identify as concerning in a novice student. It is possible, that students with an i style may spend less time self-reflecting and evaluating both learner and situational needs in favour of progressing towards an outcome. Such behaviours could potentially lead to not recognising situations that are outside of scope, that require increased support or need more objectivity and evidence-base to their approach - all of which would be highly concerning for clinical educators who are tasked with assessing student’s clinical competence.

Whilst these behaviour attributes may be alarming to a clinical educator working with a novice student, knowledge of a students’ i style could be useful for clinical educators to aid in providing opportunities to develop self-reflection strategies aligning with the Australian physiotherapy practice threshold: Role 4 *– reflective practitioner and self-directed learner* [5]. Acknowledging that reflective practice is important for all students and health practitioners to further develop, it may be particularly critical for students who wish to make changes to their behaviours at times that are most important for safe and effective clinical practice (e.g. slowing down their pace in critical care environments, despite this behaviour not being a natural tendency for someone who’s preferred behaviour style is an i). Implementation of strategies early within a clinical placement program could result in improved self-reflection, improved well-being in demanding or difficult clinical placements and an overall improved performance to achieve clinical competence. Indeed, previous research suggests that some personality and behaviour traits can be enhanced through training in self-awareness and or various mind-body exercises, leading to character building and enhanced well-being for medical students [30, 31].

Opposing to the i style, person’s with a CS style are quiet and self-controlled and show less outward energy than the average person [6]. In times of pressure, persons with a CS style are more likely to withdraw and become hesitant in making decisions [29], not wanting to engage in overly emotional or ambiguous situations. The authors of this study propose that it is these traits that educators may perceive to be a lack of knowledge, flexibility and engagement in an environment that requires decisive decisions, urgency and action and may lead a clinical educator to feel that a student displaying these traits is not clinically competent.

Interestingly, an extraversion-introversion (E-I) continuum extends diagonally through the i quadrant (extraversion) and C quadrant (introversion) of the DiSC model [6] and the two DiSC styles that are the highest predictors of clinical placement failure in physiotherapy (i and CS) are almost parallel to this continuum. Scullard & Baum [6] identify that the two dimensions of the DiSC (moderate-fast paced and skeptical-accepting) both correlate with the E-I continuum, however both styles utilize different aspects. This means that all DiSC styles will utilize some extraversion or introversion, though the styles closer aligned to the E-I continuum line will demonstrate these behaviours more readily. This suggests that the DiSC styles angled further away from the E-I continuum are styles that are more likely able to regulate these behaviour traits to adapt to the environment or situational requirements. The DiSC styles D and Si performed the best on average APP total scores across physiotherapy clinical placements and are almost 90 degrees away from the E-I continuum line. Thus, it can be inferred that those DiSC styles that have greater association with either introversion or extroversion (i.e. less adaptability along the continuum) are likely to exhibit behaviours that are predictive of unsuccessful placement outcomes. This is supported by McCombie, O’Connor and Schumacher [13] who established that extraversion is moderately desirable to both occupational therapists and physiotherapists, due to use of assertiveness required when interacting with patients and other health professionals. The same study however highlighted the importance of demonstrating variability and individual adaptation to their audience interactions.

While some DiSC styles (i and CS) were able to predict a stronger likelihood of unsuccessful grades on clinical placements (APP percentage scores), there were significant differences between DiSC styles in only two APP items. Significant differences were revealed between the DiSC styles CS and D (for APP item 3); and CS and both D and Si (for APP item 17). The differences identified for APP item 3 – *Demonstrates ethical, legal & culturally responsive practice*, within the *Professional Behaviour* domain; suggests physiotherapy students with the CS style are less likely to demonstrate these behaviours compared to their D style physiotherapy student peers. A possible rationale for this finding is associated to the CS behaviours exhibited when under pressure, such as withdrawing and becoming hesitant. Persons with the CS style tend to have an overused sense of caution, are less prone to action, find it difficult to deviate from traditional methods and are more likely to deliberate on their options [6, 29]. The CS style is also associated with a degree of passivity, letting others take control of situations [6]. Based on these characteristics, students with a CS style may have difficulty in demonstrating flexibility, urgency, decisiveness, assertiveness and potentially may not communicate or adapt to practices as readily as required. Clinical educators consequently could interpret this as the student not adapting to situations, lacking action or knowledge and relying on others, impacting on achievement of the clinical competency in the professional behaviour domain.

Physiotherapy students with the CS style also had significantly lower scores on the APP item 17 – *Progresses intervention appropriately*, within the *Intervention* domain; demonstrating lower proficiency in progressing patient interventions appropriately compared to physiotherapy students with D and Si styles. Previous research has demonstrated progression of intervention as one of the most difficult items for all physiotherapy students to perform [23]. Perhaps physiotherapy students with CS styles are likely to have greater challenges in this area as they exhibit priority behaviours consistent with stability, accuracy and support [6]. Persons with CS styles are less likely to take risks or make rapid changes, and place high priority on accuracy, taking more time refining ideas and deliberating on options before moving forward [6]. Comparatively persons with a D style have priorities of results, action and challenge [6] and commonly focus on achieving goals quickly and tend to be fast-paced. Persons with a D style tend to look at challenges as opportunities to control the outcome and therefore are more likely to progress interventions quickly working toward successful results [29]. Physiotherapy students with an Si style display behaviour priorities that focus on collaboration, support and enthusiasm [6] and they enjoy working collaboratively in their decision making, placing high importance on other people’s needs and helping them fulfill their needs [29]. The results of the present study suggest that the highest achievers in this APP item, are driven by results-oriented behaviour progressing patients’ interventions more vigorously based on their clinical reasoning (D style), or focusing on patients’ needs and concerns, using their collaboration with the patient to impact the way they would progress the patient’s care (iS). Whereas physiotherapy students with a CS style are more cautious and take a steady step-by-step approach, which clinical educators could perceive to be less than optimal and not clinically competent or efficient.

When examining the predictive relationship between the DiSC styles and APP grades for Master’s level students and Bachelor’s level students independently, the results for DiSC styles which were more likely to fail a placement were replicated in the Master’s level students however, the same statistically significant trend was not demonstrated with physiotherapy students in the Bachelor-level program. The inability to replicate the findings between students in the Master’s level and Bachelor’s level programs is likely related to the small sample of physiotherapy student participants in the Bachelor’s level program. Despite this limitation, this information remains useful to both universities and physiotherapy clinical educators in identifying physiotherapy students with DiSC styles of i and CS to provide targeted support of behaviour patterns which are more likely to result in unsuccessful outcomes during clinical placements. Importantly, this information also demonstrates that physiotherapy students across all DiSC styles have a range of APP results and students with DiSC styles other than i and CS can also potentially fail a placement.

### Limitations of the study

Although the present study provides understanding of student preferred behaviour patterns relative to their ‘Everything DiSC’ profile, including identifying those students that are more likely to fail a clinical placement and who may require targeted support to achieve success on clinical placements, it does have some limitations. Due to disproportionate sample size between undergraduate and postgraduate physiotherapy students, the identification of DiSC styles associated with failing a placement for undergraduate physiotherapy students might have been underestimated. The results investigating the differences between students based on level of study may also have been impacted by selection bias in the undergraduate cohort. Research with a larger sample may be warranted to explore these differences further. Larger sample sizes may have also revealed further differences between styles in the APP items as some additional assessment and intervention items were approaching significant differences between DiSC styles (e.g. APP Item 7 - *Conducts appropriate client-centered interview*; APP Item 13 – *Selects appropriate interventions*). Further, whilst the authors explored the impact of several potential confounders in the analysis, it is possible that there were other confounders not measured or analysed (e.g. Grade Point Average or stressors during the placements) which may have led to biased estimates of the predictor variable. Finally, it is important to acknowledge that this study is observational in nature and future research is needed to explore the effects of early identification and implementation of strategies to moderate the behaviour states of students who display preferred behaviour styles that are more likely than others to fail a five-week clinical placement in physiotherapy.

## Conclusion

This study revealed that a variety of DiSC styles are apparent in physiotherapy students with Conscientiousness (C) styles being more common. Physiotherapy students with DiSC styles Influence (i) and Conscientiousness / Steadiness (CS) were more likely in this study to fail a physiotherapy clinical placement during their studies. All physiotherapy students are required to complete clinical placements to achieve clinical competence and proficiency to graduate and become registered as safe and effective physiotherapists. Whilst most physiotherapy students can achieve passing standards by the end of a five-week placement, it is important to acknowledge that some students may have difficulty calibrating their performance to expectations in this relatively short time period but with extra time may be able to do this with or without additional support. Although all DiSC styles are thought to be equally valuable, and all individuals are a blend of all four styles, understanding physiotherapy students’ preferred behaviour styles may be useful for developing early strategies to encourage and enhance behaviour states in students that are known to result in more successful outcomes in physiotherapy clinical placement environments. Whilst preferred behaviour styles are unlikely to change over time, it is possible that some students, through reflective practice, may develop insight that will assist them to better modify their behaviour states and associated clinical performance, to meet the expectations of the clinical setting. Additionally, early awareness of a students’ DiSC style may assist both physiotherapy students and clinical educators to work collaboratively to utilize the insights from this study to moderate the behaviours that are less conducive to successful clinical placement outcomes. Although this study has established a relationship between physiotherapy students preferred behaviour styles and clinical placement grades, further research is required with a larger undergraduate physiotherapy sample size to establish if there is a difference in the relationship for undergraduate versus postgraduate students. Future research is also needed to explore the efficacy of implementing strategies early to moderate the behaviour states of students who display behaviour styles that are more likely than others to fail physiotherapy clinical placements.

## Data Availability

Currently data for this study remains stored on a locked password protected file in the organisation approving the study protocol as this was a requirement of the approving ethics committee. The datasets used and/or analysed during the current study are available from the corresponding author on reasonable request.

## Abbreviations

APC: Australian Physiotherapy Council
APP: Assessment of Physiotherapy Practice
C: Conscientiousness
CD: Conscientiousness / Dominance
CETI: Clinical Education Training Initiative
CLEI: Clinical Learning Environment Inventory
D: Dominance
DAA: Dietitians Association, Australia
DC: Dominance / Conscientiousness
Di: Dominance / Influence
HWA: Health Workforce Australia
i: Influence
iD: Influence / Dominance
iS: Influence / Steadiness
QPPC: Queensland Physiotherapy Placements Collaborative
S: Steadiness
SC: Steadiness / Conscientiousness
